# Supplementary material for: MPXV RNA-seq data provide evidence for protection of viral transcripts from APOBEC3 editing
Source: J Virol. 2026 Jan 27;100(2):e02082-25. doi: 10.1128/jvi.02082-25 (PMC12911903; doi:10.1128/jvi.02082-25)
Supplement: Supplemental material — Figures S1 to S5; Tables S1 to S3. [file jvi.02082-25-s0003.pdf]

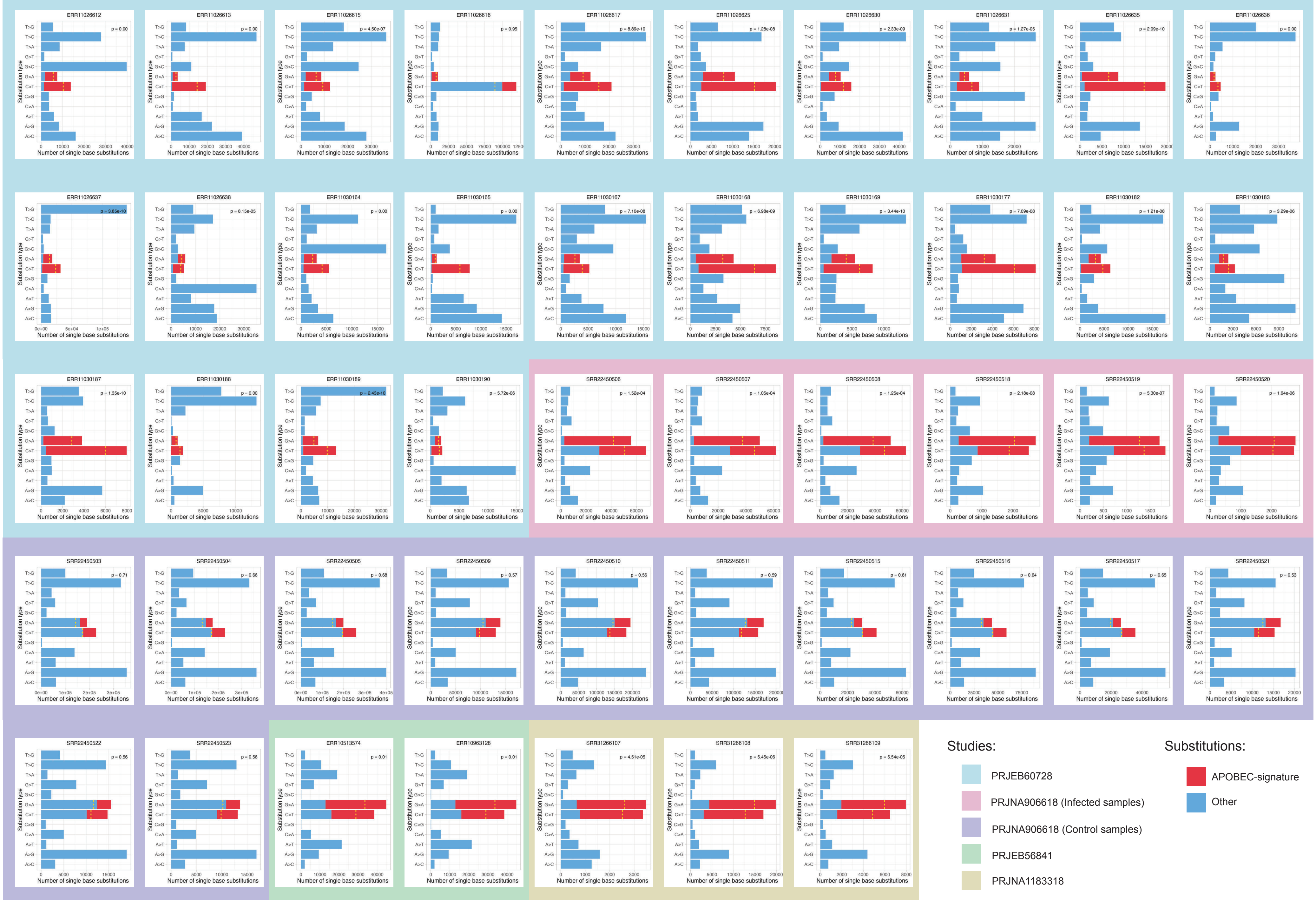

Figure S1. Distribution of observed substitutions in MPXV transcripts across substitution types. The dotted line marks the expected number of APOBEC-signature substitutions (C>T preceded by T and complementary G>A substitutions followed by A), as estimated from a beta-binomial model. The corresponding p-value indicates the significance of deviation from this expectation.

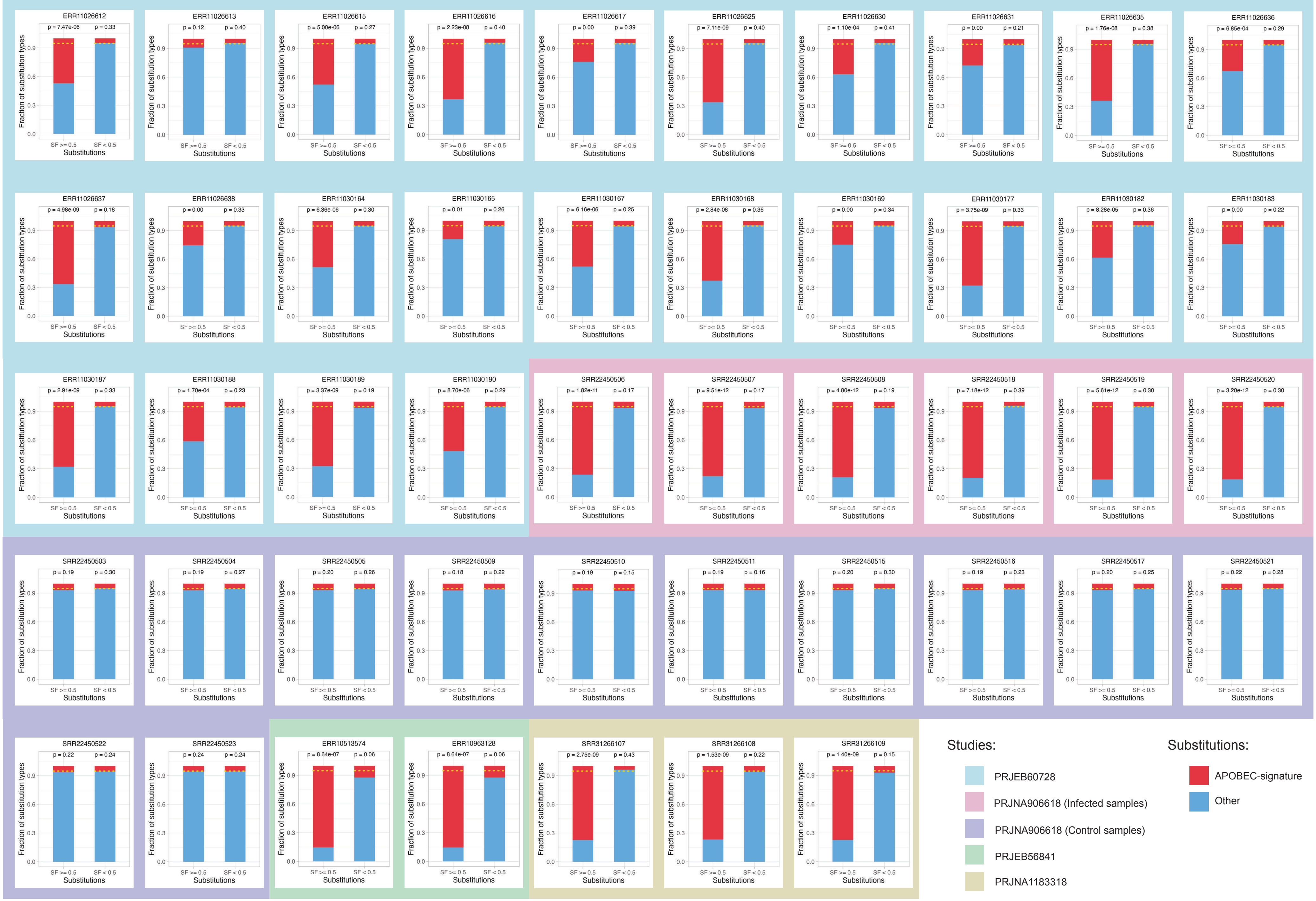

Figure S2. Enrichment analysis of APOBEC-signature substitutions across samples. Bars show the proportions of APOBEC-signature (red) and other (blue) substitutions within two substitution-frequency categories: high-frequency (SF ≥ 0.5) and low-frequency (SF < 0.5). Horizontal dashed lines represent the expected fraction of APOBEC-signature substitutions estimated from the beta-binomial model. P-values indicate the significance of deviation from this expectation for each sample.

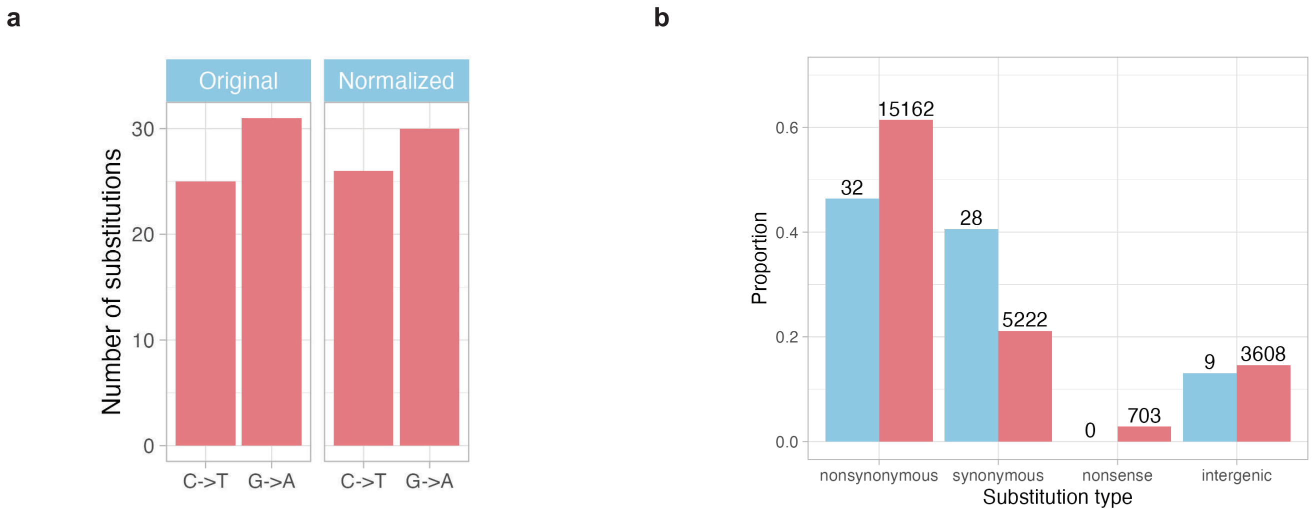

Figure S3. (a) The number of G→A substitutions identified in RNA-seq:monkey1-2 dataset remains elevated after normalization of C->T and G->A substitutions based on gene strand orientation. (b) Analysis of the effect of APOBEC-signature substitutions identified in RNA-seq:monkey1-2 dataset on the amino acid content encoded in MPXV transcripts reveals a prevalence of synonymous substitutions and a depletion of non-synonymous and nonsense substitutions.

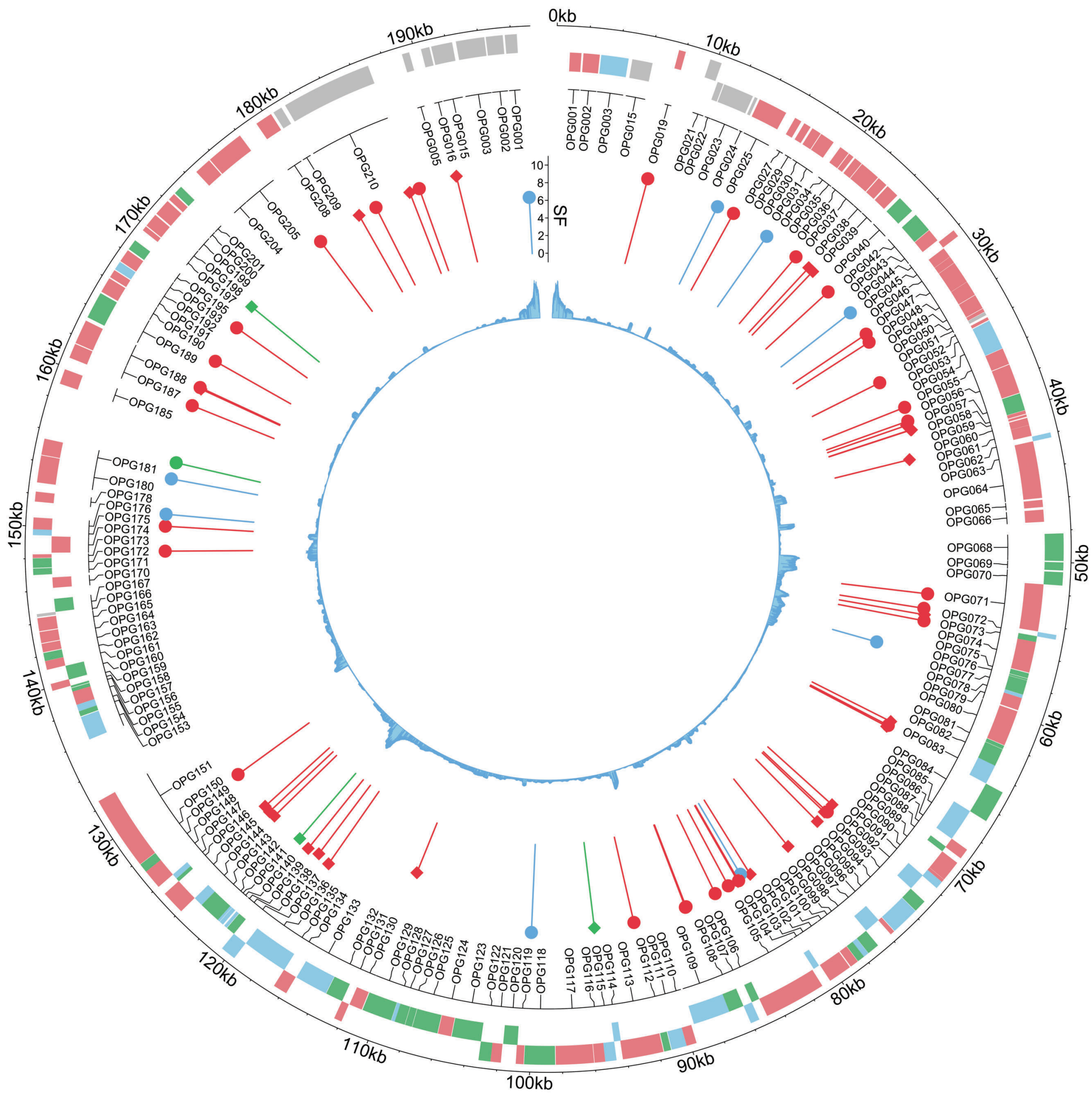

Figure S4. Genomic map of identified substitution positions in the MPXV genome from two monkey-derived projects. The outermost track shows annotated viral genes, color-coded by expression phase: early (green), middle (red), and late (blue). The next layer inward displays the positions of identified substitutions. The height of each pin reflects the frequency of the substitution. Pin color indicates substitution type: APOBEC-signature (red), extended APOBEC-signature (green), and other substitutions (blue). The pin head shape denotes strand orientation: a circle indicates a C->T substitution on the gene strand, while a square indicates the same substitution on the complementary strand. The innermost track displays the mean coverage across the genome.

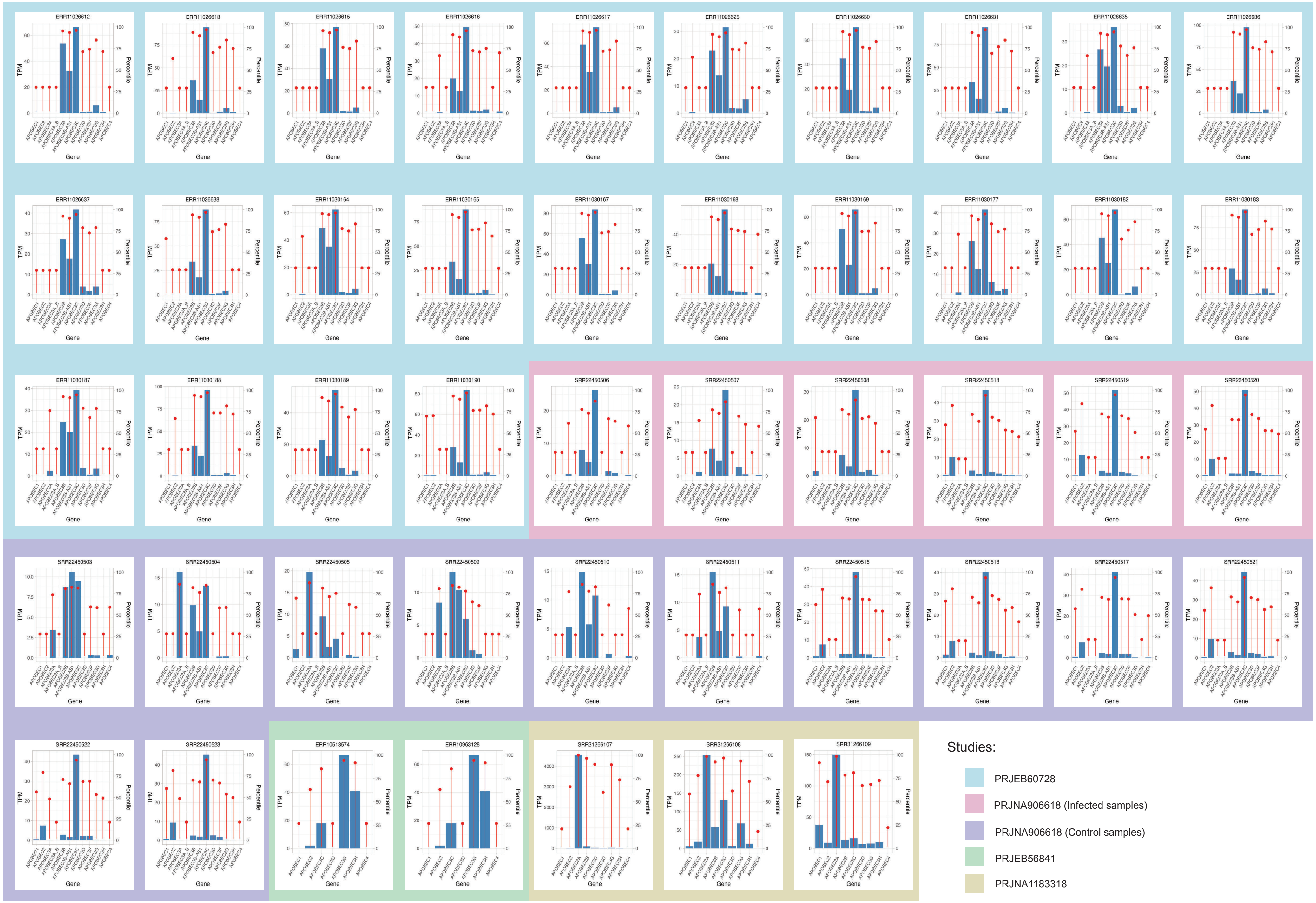

Figure S5. Each panel corresponds to a single sample and shows the expression of APOBEC genes (x-axis). Bars represent absolute transcript abundance in TPM (left y-axis). Red points denote the percentile position of each gene's TPM within the sample's full expression distribution (right y-axis), illustrating both absolute and relative expression patterns of APOBEC enzymes across the dataset.

Accession	Project title	Organism	Data type	Number of samples
PRJEB60728	Multi-omics characterization of the monkeypox virus infection	Homo sapiens	RNA-seq	24
PRJNA906618	Virological characterization of the 2022 outbreak-causing monkeypox virus using human keratinocytes and colon organdies	Homo sapiens	RNA-seq	18 (6 infected, 12 control)
PRJEB56841	Long read, high-coverage transcriptome sequencing of Monkeypox virus and CV-1 host cell line on Oxford Nanopore Technologies flow cells in different time points of infection	Chlorocebus sabaeus	RNA-seq	2
PRJNA1183318	A Penta-Component Mpox mRNA Vaccine Induced Protective Immunity in Naive and Simian Immunodeficiency Virus Infected Nonhuman Primates	Macaca fascicularis	RNA-seq	3
PRJNA845087	Illumina whole-genome sequence of Monkeypox virus in a patient traveling from Canary Islands to France	Homo sapiens	DNA-seq	4
PRJNA981509	Mpox reinfection in a vaccinated patient from Madrid (Spain)	Homo sapiens	DNA-seq	2

Table S1. Summary of sequencing projects used in this study.

<table>
<tr><th>Method description</th><th>Command line</th></tr>
<tr><td>Alignment of long RNA reads to the host, viral, and hybrid reference genomes using Minimap2</td><td><code>$minimap -t $threads -a -x splice -Y -C5 --cs --MD
-un -G 10000 $ref $fastq | $samtools sort |
$samtools view --threads $threads --write-index -b
-o "${bam}"</code></td></tr>
<tr><td>Alignment of short RNA reads to the host, viral, hybrid reference genomes using STAR</td><td><code>"${star}" --genomeDir $ref_star --runThreadN
$threads --readFilesIn $fastq --readFilesCommand
"gunzip -c" --outMultimapperOrder Random --
outSAMmapqUnique 60 --outSAMtype BAM
SortedByCoordinate --twopassMode Basic --
outFileNamePrefix "${sample_star_dir}" --outTmpKeep
All --outStd BAM_SortedByCoordinate --
outSAMunmapped Within KeepPairs --limitBAMsortRAM
1034634632 | $samtools view --threads $threads --
write-index -b -o "${bam}"</code></td></tr>
<tr><td>Alignment of short DNA reads to the host, viral, hybryd reference genomes using BWA</td><td><code>"${bwa2}" mem -R "@RG\tID:${sample}
\tPL:ILLUMINA\tLB:IDT\tSM:${sample}" "${ref}" -K
100000000 -M -Y $fastq -t 10 | $samtools sort --
write-index -m 500M -T "${sample_output_dir}/$
{sample}.sort" --threads 10 -o "${bam}"</code></td></tr>
<tr><td>Filtering of viral reads aligned to the hybrid genome from the BAM file</td><td><code>$samtools view --threads $threads -P $hybryd_bam --
write-index -b -o "${virus_bam_from_hybrid_bam}" “$
{virus_contig}”</code></td></tr>
<tr><td>Extraction of unmapped reads from the host-aligned BAM file</td><td><code>$samtools view --threads $threads -f 4 $host_bam --
write-index -b -o "${virus_bam_from_host_bam}"</code></td></tr>
<tr><td>Extraction of reads mapped to the viral genome from the BAM file</td><td><code>$samtools view --threads $threads -F 4 $virus_bam
--write-index -b -o "${virus_bam_from_virus_bam}"</code></td></tr>
<tr><td>Generation of FASTQ files from the filtered BAM file</td><td><code>$samtools collate --threads $threads -u -O "$
{filtered_bam}" | $samtools fastq --threads
$threads -n -1 "${virus_fastq1_from_filtered_bam}"
-2 "${virus_fastq2_from_filtered_bam}" -0 "$
{virus_fastqS_from_filtered_bam}" -s "$
{virus_fastqO_from_filtered_bam}"</code></td></tr>
<tr><td>Variant calling using BCFtools with quality thresholds on mapping quality (MQ) and base quality (BQ)</td><td><code>$bcftools mpileup --threads $threads --min-MQ $MQ
--min-BQ $BQ --max-depth 1000000 --skip-indels -f
$virus_ref -Ou "${realigned_bam}" | $bcftools norm
--threads $threads -m -snps --keep-sum AD --force |
grep -v "*" | $bcftools +fill-tags -- -t VAF |
$bcftools view --threads $threads --write-index -Oz
-o "${sample_output_dir}/${sample}_bcftools_MQ${MQ}
BQ${BQ}.vcf.gz"</code></td></tr>
</table>

Table S2. Command-line parameters used in the bioinformatic pipeline.

Project	Sample	P-value for A->G/T->C enrichment	P-value for A->G transcription strand bias	P-value for A->G enrichment in dsRNA
PRJEB56841	ERR10513574	0.346319803	0.147297779	0.617637517
PRJEB56841	ERR10963128	0.346294097	0.147297779	0.617637517
PRJEB60728	ERR11026612	0.073200495	0.410432918	0.817371511
PRJEB60728	ERR11026613	0.009469853	0.754351972	0.85501291
PRJEB60728	ERR11026615	0.034027921	0.722644347	0.946954614
PRJEB60728	ERR11026616	0.424172904	0.761075434	0.840911218
PRJEB60728	ERR11026617	0.030523079	0.750361712	0.988738199
PRJEB60728	ERR11026625	0.018484952	0.593115545	0.995329466
PRJEB60728	ERR11026630	0.032725963	0.559860267	0.999617289
PRJEB60728	ERR11026631	0.026255669	0.189740388	0.816773513
PRJEB60728	ERR11026635	0.035959919	0.514115832	0.989860909
PRJEB60728	ERR11026636	0.000648505	0.4356497	0.999996817
PRJEB60728	ERR11026637	0.40402067	0.462226706	0.290630913
PRJEB60728	ERR11026638	0.066357285	0.387051522	0.882820375
PRJEB60728	ERR11030164	0.0716565	0.403650116	0.990125445
PRJEB60728	ERR11030165	0.006488889	0.811104849	0.921939143
PRJEB60728	ERR11030167	0.037026653	0.592913645	0.467159912
PRJEB60728	ERR11030168	0.101237814	0.697702333	0.468069161
PRJEB60728	ERR11030169	0.028515134	0.727842909	0.997990244
PRJEB60728	ERR11030177	0.019130973	0.472563816	0.99355232
PRJEB60728	ERR11030182	0.030961895	0.672331583	0.987006157
PRJEB60728	ERR11030183	0.029594589	0.192871361	0.934309925
PRJEB60728	ERR11030187	0.039057731	0.429163792	0.996358089
PRJEB60728	ERR11030188	0.000531134	0.262018374	0.999967765
PRJEB60728	ERR11030189	0.259227462	0.469875758	0.172573194
PRJEB60728	ERR11030190	0.067285935	0.389125748	0.998884165
PRJNA906618	SRR22450506	0.614348728	0.611560639	0.998185191
PRJNA906618	SRR22450507	0.615873168	0.675367242	0.87761607
PRJNA906618	SRR22450508	0.625168793	0.705321376	0.779027949
PRJNA906618	SRR22450518	0.142791104	0.440672968	0.576472215
PRJNA906618	SRR22450519	0.178995282	0.371218209	0.826409476
PRJNA906618	SRR22450520	0.163528049	0.466670893	0.994254253
PRJNA1183318	SRR31266107	0.124014913	0.3088301	0.893855178
PRJNA1183318	SRR31266108	0.088349699	0.345706409	0.998671215
PRJNA1183318	SRR31266109	0.057846583	0.326231248	0.89400051

Table S3. Statistical evaluation of A→G substitutions across samples. The table reports p-values for overall A→G/T→C enrichment, bias toward the transcribed strand, and enrichment of A→G substitutions within predicted double-stranded RNA (dsRNA) regions, which are characteristic features of ADAR-mediated RNA editing.
